
JOURNAL INFORMATION
==============================
NLM Title Abbreviation: ScientificWorldJournal
Journal ID: ScientificWorldJournal
NLM Journal ID: 101131163
Journal ID: TSWJ

The Scientific World Journal

ISSN: 2356-6140
EISSN: 1537-744X
Publisher: Wiley

ARTICLE INFORMATION
==============================
PMCID: PMC6009402
PMID: 29973855
DOI: 10.1100/tsw.2002.297
Article ID: 984042
Article version: 1
Subjects: Editorial

Selected Short Communications from the International Conference on Environmental Concerns and Emerging Abatement Technologies, Beijing, China, 9–12 October, 2001

Wang Zijian *
SKLEAC, Research Center for Eco-Environmental Sciences, Shuangqing Rd. 18, Beijing 100085, China
*Zijian Wang: wangzj@mail.rcees.ac.cn
Publication date: 2002
Electronic publication date: 2002 May 22
Volume: 2
First page: 1433
Last page: 1434
Received 2002 Apr 8; Accepted 2002 Apr 10
Copyright: Copyright © 2002 Zijian Wang.
Copyright year: 2002
License: This is an open access article distributed under the Creative Commons Attribution License, which permits unrestricted use, distribution, and reproduction in any medium, provided the original work is properly cited.
License URL: https://creativecommons.org/licenses/by/3.0/

Keywords: environmental pollution and monitoring, ecotoxicology and risk assessment, abatement technologies, cleaner production technologies, policy issues, environmental economics, modeling

==============================
**Editorial**
The International Conference on Environmental Concerns and Emerging Abatement Technologies 2001: Collection of Short Communications
*TheScientificWorldJOURNAL* (2002) 2, 1433–1434
ISSN 1537-744X; DOI 10.1100/tsw.2002.297

### Selected Short Communications from the International Conference on Environmental Concerns and Emerging Abatement Technologies, Beijing, China, 9–12 October, 2001

Zijian Wang (Guest Editor)

*SKLEAC, Research Center for Eco-Environmental Sciences, Shuangqing Rd. 18, Beijing 100085, China*

E-mail: wangzj@mail.rcees.ac.cn

*Received April 8, 2002; Accepted April 10, 2002; Published May 22, 2002*

---

**KEY WORDS:** environmental pollution and monitoring, ecotoxicology and risk assessment, abatement technologies, cleaner production technologies, policy issues, environmental economics, modeling

**DOMAINS:** environmental chemistry, atmospheric systems, environmental management and policy, terrestrial environmental toxicology, freshwater systems, waste management policy, environmental monitoring

---

In October 2001, a 4-day International Conference on Environmental Concerns and Emerging Abatement Technologies was held in Beijing, China. The conference brought together about 450 participants from 20 countries and 303 papers were collected in the proceeding of the conference. The topics covered in the conferences included environmental pollution and monitoring, ecotoxicology and risk assessment, abatement technologies, cleaner production technologies, policy issues and modeling. From these topics, 12 plenary, 36 session-keynote, 84 oral presentations, as well as more than 100 posters were presented. Many renowned scientists from the world of environmental science and technology where invited to address topics such as on atmospheric chemistry and climate change (by P. Crutzen), environmental biotechnology (by J. Paques and W. Verstraete), ecotoxicological risk assessment (by H. Eijsackers), chemical speciation and bioavailability (by H. Allan), cleaner production (by J. Dillon), as well as environmental economics (by E. van Ierland).

The conference provided a good forum to exchange ideas between representatives from developed and developing countries, especially related to China, where social, cultural, economical, geographical, and scientific background are different. The discussions on the differences in developing stages, scientific thinking, and social and cultural discrepancies proved meaningful as it resulted in a common agreement that environmental policy and technologies

©2002 with author.

cannot simply be copied or transferred from one country to another. For example, in setting priorities in environmental research, papers from scientists of the developed countries focused on public health, ecosystems safety, and remediation of the polluted environment, while most of the papers from Chinese contributors focused on environmental pollution surveys and end-pipe treatment technologies. Also, discussions on policy issues and models, especially models based on the concept of environmental economy, were well debated in several sessions. China is undergoing an economic transformation process and merging environmental management into market economy will certainly be a big challenge for scientists and decision makers in future years. Exchange of ideas between people from different countries could help lead them in the right direction.

I am indebted to TheScientificWorld and in particular Dr. Wendy Ockenden and Sandra Jenkins for giving our participants the opportunity to publish their papers in this e-journal. This Themed Issue of *TheScientificWorldJOURNAL* presents fully peer-reviewed short communications from original papers contributed to the conference. Readers may also find further conference papers in the special issue of the *Journal of Environmental Science and Health, Part A – Toxic/Hazardous Substances & Environmental Engineering,* Vol. 37, No. 4 (Dekker Publishers).

**This article should be referenced as follows:**

Wang, Z. (2002) Selected short communications from the International Conference on Environmental Concerns and Emerging Abatement Technologies, Beijing, China, 9–12 October, 2001. In The International Conference on Environmental Concerns and Emerging Abatement Technologies 2001: Collection of Short Communications. *TheScientificWorldJOURNAL* **2**, 1433–1434.

### BIOSKETCH

**Zijian Wang** is a full-time professor at the Research Center for Eco-Environmental Sciences (RCEES), Chinese Academy of Sciences (CAS). He was the general secretary of the International Conference on Environmental Concerns and Emerging Abatement Technologies, held in Beijing, October 9–12, 2001. As a researcher, he has published more than 180 peer-reviewed scientific papers in the fields of environmental chemistry and ecotoxicology within 52 international journals.
